# Stable Transgenic Mouse Strain with Enhanced Photoactivatable Cre Recombinase for Spatiotemporal Genome Manipulation

**DOI:** 10.1002/advs.202201352

**Published:** 2022-10-20

**Authors:** Huiying Li, Yingyin Wu, Yuhao Qiu, Xinru Li, Yuting Guan, Xiya Cao, Meizhen Liu, Dan Zhang, Sijie Huang, Longnian Lin, Lijian Hui, Xueyun Ma, Mingyao Liu, Xueli Zhang, Liren Wang, Dali Li

**Affiliations:** ^1^ Shanghai Frontiers Science Center of Genome Editing and Cell Therapy Shanghai Key Laboratory of Regulatory Biology and School of Life Sciences East China Normal University Shanghai 200241 China; ^2^ Southern Medical University Affiliated Fengxian Hospital Shanghai 201499 China; ^3^ Key Laboratory of Brain Functional Genomics (Ministry of Education) Institute of Brain Functional Genomics East China Normal University Shanghai 200062 China; ^4^ State Key Laboratory of Cell Biology CAS Center for Excellence in Molecular Cell Science Shanghai Institute of Biochemistry and Cell Biology University of Chinese Academy of Sciences Chinese Academy of Sciences Shanghai 200031 China

**Keywords:** cell ablation, cell labeling, optogenetics, photoactivatable Cre recombinase

## Abstract

Optogenetic genome engineering is a powerful technology for high‐resolution spatiotemporal genetic manipulation, especially for in vivo studies. It is difficult to generate stable transgenic animals carrying a tightly regulated optogenetic system, as its long‐term expression induces high background activity. Here, the generation of an enhanced photoactivatable Cre recombinase (ePA‐Cre) transgenic mouse strain with stringent light responsiveness and high recombination efficiency is reported. Through serial optimization, ePA‐Cre is developed to generate a transgenic mouse line that exhibits 175‐fold induction upon illumination. Efficient light‐dependent recombination is detected in embryos and various adult tissues of ePA‐Cre mice crossed with the Ai14 tdTomato reporter. Importantly, no significant background Cre activity is detected in the tested tissues except the skin. Moreover, efficient light‐inducible cell ablation is achieved in ePA‐Cre mice crossed with Rosa26‐LSL‐DTA mice. In conclusion, ePA‐Cre mice offer a tightly inducible, highly efficient, and spatiotemporal‐specific genome engineering tool for multiple applications.

## Graphical abstract

In this study, the generation of an enhanced photoactivatable Cre recombinase (ePA‐Cre) transgenic mouse strain which enables stringent light‐induced cell depletion and genome manipulation is reported. The superior stringency of the ePA‐Cre mouse makes the model a powerful tool for efficient, spatiotemporal, highly tunable, and noninvasive genome engineering for further biological applications.

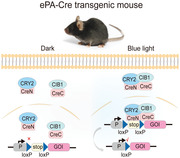

## 1 Introduction

Inducible genome engineering is a powerful and critical genetic technology for not only basic research but also industrial applications.^[^ ^1^ ^]^ The inducible Cre‐loxP system, which was derived from the P1 bacteriophage, is the most widely used tool for manipulating DNA recombination and gene expression.^[^ ^2^ ^]^ Although several chemically induced Cre‐loxP systems, such as tamoxifen‐inducible Cre‐ER,^[^ ^3^ ^]^ doxycycline‐inducible P_tet_‐Cre,^[^ ^4^ ^]^ rapamycin‐inducible DiCre,^[^ ^5^ ^]^ and trimethoprim‐inducible DD‐Cre,^[^ ^6^ ^]^ have been developed, the relatively slow kinetics upon induction and low spatial resolution limit the applications of these systems in a highly spatiotemporal manner.

Recently, several photoactivatable Cre recombinase systems based on the reassembly of split Cre fragments have been developed, which are particularly attractive due to their spatiotemporal precision, high tunability, and noninvasiveness.^[^ ^7^ ^]^ These systems are divided into two groups: one group of systems regulates at the transcriptional level, and the other regulates at the posttranslational level. The representative transcriptional control system is the far‐red‐light inducible Cre‐loxP system (FISC) based on the bacterial photoreceptor BphS, which offers high penetration capacity and strong transcriptional induction but requires continuous illumination for several hours.^[^ ^8^ ^]^ As it contains quite complicated components, it is not feasible for generating transgenic animals. Due to their prompt light responsiveness, posttranslational regulation systems are more widely used for generating photoactivatable Cre recombinase. In this approach, inactive split Cre fragments are directly tethered to various individual subunits of light sensing components that dimerize upon illumination to reconstitute a functional recombinase. Among them, the red‐light‐dependent Cre‐loxP system (CreLite) enables multispectral cell labeling in zebrafish embryos.^[^ ^9^ ^]^ However, CreLite, based on the PhyB‐PIF6 system, requires the addition of an exogenous phycocyanobilin chromophore, which limits its in vivo applicability, especially in higher animals. Blue light‐induced dimerization modules, such as cryptochrome 2 (CRY2), magnets, vivid (VVD), and AsLOV2, have been used to develop photoactivatable Cre recombinases without the need for exogenous chromophores.^[^ ^10^, ^11^, ^12^, ^13^, ^14^ ^]^ Among them, improved versions of the CRY2‐CIB1 split Cre system, named PA‐Cre2.0 and the Magnets split Cre system, are widely used due to their rapid and efficient light responsiveness. These two systems have been demonstrated to have successful applications in multiple mouse tissues by delivery of plasmids or adeno‐associated viruses expressing photoactivatable Cre recombinases.^[^ ^12^, ^15^ ^]^ However, these transient transgenic models have several limitations, such as exogenous DNA delivery that can only reach limited tissue/cell types with highly variant transduction efficiency, difficulty in modulating embryos, and varied expression levels across individual animals due to the injection skill of the experimenter. Stable PA‐Cre transgenic mice could overcome the above disadvantages. Two reports have demonstrated the generation of stable transgenic mice with two versions of the Magnets‐based PA‐Cre, but the old version of PA‐Cre transgenic mice exhibited significant leaky activation,^[^ ^16^ ^]^ while the performance of the PA‐Cre 3.0 transgenic mice has not been carefully characterized.^[^ ^17^ ^]^ A high‐performance, stable PA‐Cre transgenic mouse strain is highly desirable for convenient, efficient, and prompt spatiotemporal genetic engineering in more cell types.

In this study, we optimized a CRY2‐CIB1‐based PA‐Cre2.0 construct and generated a stable transgenic mouse line harboring the ePA‐Cre system that enables high induction (up to 175‐fold). Using an endogenous reporter strain, the ePA‐Cre system functions efficiently and stringently in multiple tissues as well as in embryos. The ePA‐Cre mouse achieved spatiotemporally efficient cell ablation when crossed with the Rosa26‐LSL‐DTA mouse. The ePA‐Cre mouse is a tightly controlled genome engineering tool that can modulate DNA recombination efficiently in a spatiotemporal‐specific manner.

## 2 Results

### 2.1 Evaluation and Optimization of PA‐Cre Constructs for Transgenesis

**Figure 1:**
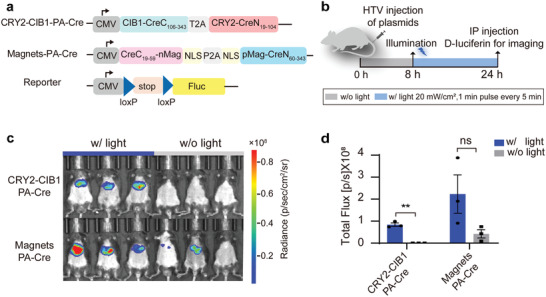
Comparison of light‐dependent recombination mediated by CRY2‐CIB1‐PA‐Cre and Magnets‐PA‐Cre in vivo. a) Constructs of two photoactivatable recombination and firefly luciferase reporters (loxP‐stop‐loxP‐Fluc). b) Schematic diagram of the in vivo detection procedure for light‐dependent recombination activity in mice. CRY2‐CIB1‐PA‐Cre/Magnets‐PA‐Cre and their reporter plasmids were delivered through the HTV. Injected mice were illuminated with blue light (w/ light) for 16 h (20 mW cm^−2^, 1 min pulse every 5 min) or without blue light (w/o light) stimulation. The luciferase activity was determined 24 h after injection. c) Bioluminescence images of the mice injected with CRY2‐CIB1‐PA‐Cre/Magnets‐PA‐Cre and loxP‐stop‐loxP‐Fluc reporter plasmids at a ratio of 1: 1. d) Quantification of the bioluminescence images presented in (c). The results are shown as the mean ± s.d.; *n* = 3 mice. Statistical analysis was calculated by Student's *t*‐test; **P < 0.01 versus control, n.s., not significant.

**Figure 2:**
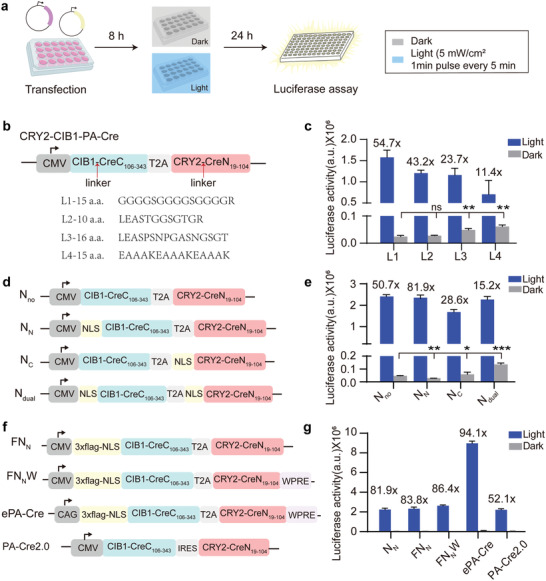
Optimization of transgene expression cassette. a) Procedure diagram of PA‐Cre induced luciferase reporter assay. HEK‐293T cells were transfected with the indicated version of CRY2‐CIB1‐PA‐Cre along with a Gaussia luciferase reporter (loxP‐stop‐loxP‐Gluc) and exposed to blue light (5 mW cm^−2^, 1 min pulse every 5 min) 8 h post‐transfection or kept in the dark. Luciferase activity was determined 24 h after the first illumination. b) Schematic of four different linkers (L1‐L4) between CIB1/CRY2 and split Cre recombinase. c) Luciferase reporter assays for evaluating the function of CRY2‐CIB1‐PA‐Cre with variant linker sequences. d) Schematic of NLS localization in the N‐terminus (N_N_), C‐terminus (N_C_), and both termini (N_dual_) of CRY2‐CIB1‐PA‐Cre, and “N_no_” for “no NLS.” e) Luciferase reporter assay for examining the effects of NLS fusion shown in (d). f) Schematic of enhanced versions of CRY2‐CIB1‐PA‐Cre. FN_N_: 3× flag tag was added on the basis of N_N_; FN_N_W: WPRE was added on the basis of FN_N_; ePA‐Cre: CMV promoter was replaced by CAG on the basis of FN_N_W. g) Luciferase reporter assays for evaluating the effect of the enhanced versions shown in (f). In (b–g), the results are shown as the mean ± s.d.; *n* = 3 independent experiments. Statistical analysis was calculated by Student's *t*‐test; *P < 0.05, **P < 0.01, ***P < 0.001 versus control. n.s., not significant.

To generate a stable PA‐Cre transgenic mouse that is highly sensitive to light with low background, PA‐Cre2.0 and Magnets‐PA‐Cre were leveraged as candidates due to their elegant performance in vivo.^[^ ^12^, ^15^ ^]^ To increase the activity of PA‐Cre2.0, we optimized the construct by replacing the internal ribosome entry site (IRES) element with self‐cleaving peptides (Figure S1a, Supporting Information) since the IRES element usually impairs downstream gene expression.^[^ ^18^ ^]^ Using a Gaussia luciferase reporter (loxP‐stop‐loxP‐Gluc), it was demonstrated that the usage of T2A peptide to link N‐terminus Cre (CreN) and C‐terminus Cre (CreC) exhibited higher‐fold induction compared with P2A counterpart peptide (50.6‐fold versus 20.4‐fold) (Figure S1b, Supporting Information). Although the actual luciferase activity was comparable, a higher induction fold indicates lower background or leakage activity of the PA‐Cre system. Next, the variants of PA‐Cre2.0 named CRY2‐CIB1‐PA‐Cre and Magnets‐PA‐Cre were directly compared in vivo (**Figure** **1**a). The Cre‐inducible firefly luciferase reporter (loxP‐stop‐loxP‐Fluc) was co‐delivered with each of the PA‐Cre systems through hydrodynamic tail vein (HTV) injection.^[^ ^19^ ^]^ The mice were illuminated with blue light 8 h post‐injection or without blue light stimulation, and the luciferase activity was examined 24 h after injection (Figure 1b). Although CRY2‐CIB1‐PA‐Cre showed lower recombination activity, almost no leakage was detected without illumination, suggesting that it was more stringent than Magnets‐PA‐Cre in vivo (Figure 1c,d). The CRY2‐CIB1‐PA‐Cre system was used for further optimization (**Figure** **2**a), since the high background activity of Magnets‐PA‐Cre might result in significant leakage effects, as previously reported.^[^ ^16^ ^]^

To further optimize the construct, four different linkers (L1‐L4) separating the split Cre fragments and CRY2 or CIB1 were tested (Figure 2b). The construct with the L1 linker ((GGGGS)_2_GGGGR) used in a previous study^[^ ^11^ ^]^ showed the highest induction (Figure 2c). Next, the nuclear localization signal sequences (NLSs) were evaluated (Figure 2d). We found that insertion of an NLS at the N‐terminus of CIB1‐CreC significantly reduced the background PA‐Cre activityapproximately 60% and maintained comparable efficiency with the original version in response to illumination, leading to an increase in the induction rate from 50.7 to 81.9‐fold (Figure 2e). Through fluorescent microscopy analysis, we found that NLS‐CIB1‐CreC was localized in the nucleus with or without illumination, while CRY2‐CreN displayed nuclear‐exporting activity upon illumination (Figure S2, Supporting Information). Although we did not know the exact reason why NLS fused to CIB1‐CreC decreased the background activity of ePA‐Cre, it was possible that additional NLS made CIB1‐CreC more concentrated and exclusive in the nucleus to decrease the chance of CIB1 to bind with CRY2 component, since tethering the NLS to CRY2‐CreN portion dramatically increased the background activity (Figure 2e). Moreover, to enhance the mRNA stability or expression level of ePA‐Cre, the woodchuck hepatitis virus posttranscriptional regulatory element (WPRE)^[^ ^20^ ^]^ and CAG promoter were employed and tested (Figure 2f). To facilitate the monitoring of PA‐Cre expression in vitro and in vivo, a 3× flag tag was also incorporated into the construct. As a result, the construct ePA‐Cre showed the highest induction rate, which was four fold higher than that previously reported for PA‐Cre2.0^[^ ^11^ ^]^ (Figure 2g), suggesting ePA‐Cre was a tightly controlled and highly efficient transgenic vector in vitro.

### 2.2 Characterization of ePA‐Cre Constructs through a Transient Transgenic Approach

**Figure 3:**
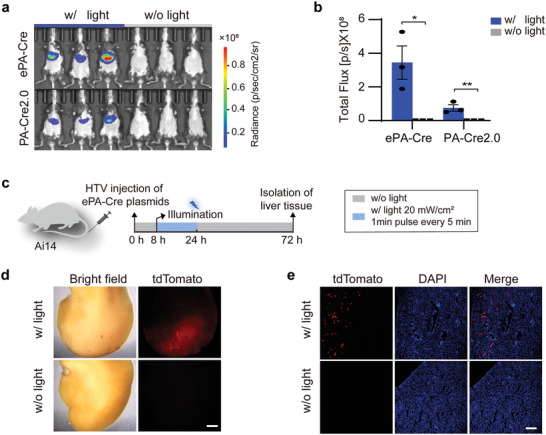
Validation of the ePA‐Cre system in vivo. a) Representative bioluminescence images of mice injected with ePA‐Cre/PA‐Cre2.0 and loxP‐stop‐loxP‐Fluc reporter plasmids at a ratio of 1:1. b) Quantification of the bioluminescence intensity of the ePA‐Cre and PA‐Cre2.0 groups presented in (a). The mice were treated with the indicated procedure in Figure 1b. The results are shown as the mean ± s.d.; *n* = 3 mice. Statistical analysis was calculated by Student's *t*‐test; *P < 0.05, **P < 0.01 versus control. c) Schematic diagram of the in vivo validation of the ePA‐Cre activity procedure in Ai14 mice. ePA‐Cre plasmids were delivered through the HTV. Injected mice were illuminated with blue light (w/ light) for 16 h (20 mW cm^−2^, 1 min pulse every 5 min) or without blue light (w/o light) stimulation. The livers were harvested 72 h after injection. d) Bright‐field and fluorescence images of the livers from ePA‐Cre‐treated Ai14 mice using a stereomicroscope. Scale bars, 2 mm. e) Representative fluorescent tdTomato (red) images of liver sections from ePA‐Cre‐treated Ai14 mice. DAPI (blue) was used to indicate the stained nucleus. Scale bar, 500 µm.

To verify ePA‐Cre in vivo, plasmids expressing ePA‐Cre or PA‐Cre2.0 along with the loxP‐stop‐loxP‐Fluc reporter were systemically delivered into mouse livers via HTV injection. Consistent with the in vitro results, ePA‐Cre induced higher luciferase activity (fivefold) than PA‐Cre2.0 in vivo (**Figure** **3**a,b). Next, ePA‐Cre plasmids were delivered to endogenous Ai14 reporter mice (Figure 3c), whose tdTomato gene expression was activated after Cre‐induced recombination. Analysis of isolated livers indicated that tdTomato expression was present in the livers of the illuminated mice (Figure 3d,e), suggesting that ePA‐Cre was capable of catalyzing endogenous gene recombination. Taken together, these results suggested that the ePA‐Cre system functions remarkably through a transient transgenic approach in vivo.

### 2.3 Generation of the ePA‐Cre Mouse Model

**Figure 4:**
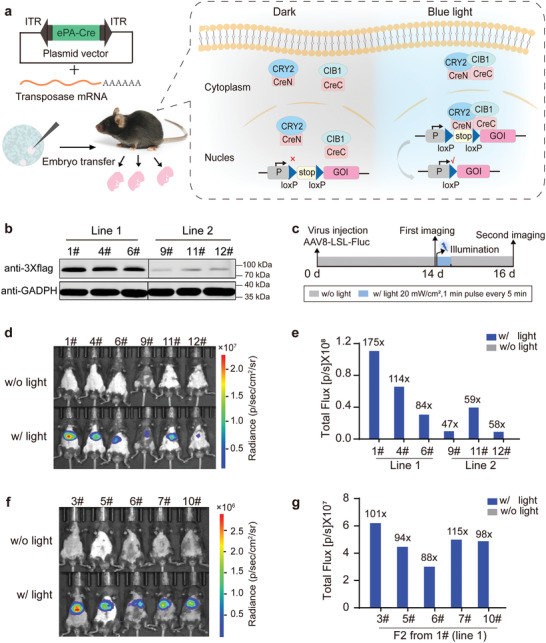
Generation and characterization of ePA‐Cre mice. a) Schematic diagram of ePA‐Cre mouse generation via SB‐mediated gene transfer and its working principle. The ePA‐Cre cassette was cloned between the inverted terminal repeats (ITRs) of the transposon donor plasmid and integrated at a chromosomal site by the transposase. The transcriptional stop cassette flanked by two loxP sites was cut by blue‐light‐induced ePA‐Cre to allow gene of interest (GOI) expression. b) Representative immunoblotting images of the ePA‐Cre expression level from three different offspring of line 1 and line 2. Lysates were derived from mouse tail fibroblast cells. CIB1‐CreC_106‐343_ was immunoblotted with an anti‐flag antibody (top). GAPDH protein was used as loading control (bottom). c) Schematic diagram of the functional procedure using the offspring of line 1 and line 2. loxP‐stop‐loxP‐Fluc (AAV‐LSL‐Fluc) plasmids were packaged into a hepatocyte‐tropic AAV8 virus and delivered into mice through tail vein injections. The luciferase activity was first determined 2 weeks after injection. The mice were then illuminated with blue light (20 mW cm^−2^, 1 min pulse every 5 min for 16 h), and the second luciferase assay was performed 2 days after illumination. d) Bioluminescence images of mice injected with AAV8 virus before and after illumination. e) Quantification of the bioluminescence intensity is presented in (d). f) Bioluminescence images of the F2 generation mice injected with AAV8 virus before and after illumination. The mice were treated as the procedure in (c). The luciferase activity was first determined 2 weeks after injection. The mice were then illuminated with blue light (20 mW cm^−2^, 1 min pulse every 5 min for 30 min), and the second luciferase assay was performed 2 days after illumination. g) Quantification of the bioluminescence intensity of the F2 generation in (f).

As ePA‐Cre exhibited superior performance in all tested constructs, it was used to generate a transgenic mouse strain under a ubiquitously expressed promoter by *Sleeping Beauty* (SB) transposon‐mediated gene transfer^[^ ^21^ ^]^ (**Figure** **4**a). Two independent mouse lines were established from distinct founders. Western blotting data showed that the protein level of ePA‐Cre (3× flag‐NLS‐CIB1‐CreC) in F1 fibroblasts derived from line 1 was much higher than that of line 2 (Figure 4b). As the protein level does not fully reflect the function of transgenes in the animals, we performed functional analysis to select a better transgenic line. Three F1 mice from each founder were injected with AAV8 virus carrying the loxP‐stop‐loxP‐Fluc reporter (Figure 4c). Two weeks after injection, no luciferase activity was detected in the mouse abdomen before illumination (Figure 4d, top). These mice were then exposed to blue light, and robust luciferase activity was detected, especially in the progenies from line 1 (Figure 4d, bottom). Among them, F1‐1# was selected for further breeding due to its high efficiency and stringent control of recombination activity, which induced the highest luciferase activity (175‐fold induction) compared with the unilluminated condition (Figure 4e).

To determine the integration site of ePA‐Cre in the genome, F1‐1# mouse was subjected to whole genome sequencing, which revealed that a single‐copy of ePA‐Cre was integrated into intron 1 of *Notch2* gene (Chr3 M98016848‐98, 016849). No other integration site or any other insertions of plasmid backbone sequences were detected (Figure S3, Supporting Information). Since the size of intron 1 was relatively large (33.956 kb in length) and the ePA‐Cre integration site was not adjacent to the splicing donor or acceptor site, we speculated that the transcription of *Notch2* might not be affected. qRT‐PCR analysis of multiple tissues from ePA‐Cre mice revealed that the expression of *Notch2* as well as its downstream target genes were not affected, suggesting that the transgene did not influence endogenous *Notch2* gene expression (Figure S4, Supporting Information). To further reduce the risk of interfering with *Notch2* expression, we used heterozygous ePA‐Cre transgene mice in the following experiments. The heterozygous ePA‐Cre transgene mice were fertile and had normal litter sizes. In addition, the mice presented no cellular morphological abnormalities in various tissues (Figure S5a, Supporting Information) and no electrophysiological perturbations of neurons (Figure S5b–f, Supporting Information), indicating that constitutive ePA‐Cre expression had no adverse effects. ePA‐Cre‐mediated recombination was achieved in the progenies of F1‐1# mice following exposure to blue light for only 30 min. Luciferase activity was detected only after blue light illumination, and all F2 mice showed average of approximately 100‐fold induction of luciferase activity (Figure 4f,g). The data above showed that a germline transmissible ePA‐Cre transgenic mouse strain was successfully generated, and the following experiments were performed in F4 and their progenies.

### 2.4 Highly Efficient and Stringent Light‐Inducible Recombinase Activity in ePA‐Cre Mice

**Figure 5:**
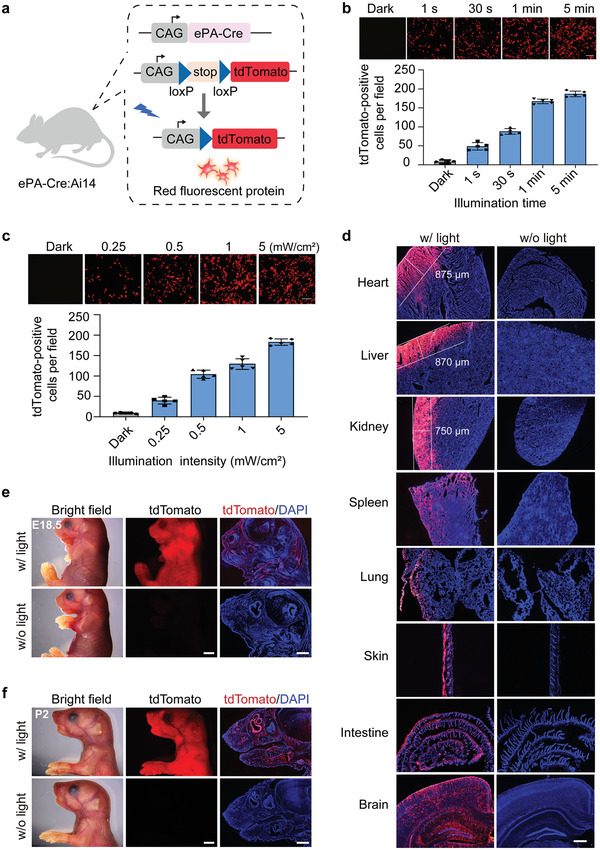
Light‐dependent recombinase activity in the ePA‐Cre: Ai14 mice. a) Schematic diagram of light‐dependent activation of the tdTomato reporter in ePA‐Cre: Ai14 mice. The transcriptional stop cassette flanked by two loxP sites was cut by ePA‐Cre upon blue‐light illumination, and the expression reporter gene tdTomato was stimulated. The following experiments were conducted in double‐heterozygous ePA‐Cre: Ai14 mice. b) Exposure time‐dependent ePA‐Cre activity. Fibroblasts isolated from mouse tail were illuminated for the indicated times (5 mW cm^−2^, continuous). c) Exposure intensity‐dependent ePA‐Cre activity. Fibroblasts isolated from mouse tail were illuminated for 1 min at the indicated intensity. In (b) and (c), fluorescent tdTomato images were observed 24 h after illumination. Scale bars, 200 µm. The graph at the bottom represents the quantification of tdTomato‐positive cells per field under the indicated conditions. The results are shown as the mean ± s.d., *n* = 5. d) Representative fluorescence images of various tissue sections from light‐dependent, genetically labeled mice. Adult mice were illuminated with blue light (w/ light) (60 mW cm^−2^, 1 min pulse every 5 min) for 48 h or without blue light (w/o light) stimulation, and tissues were harvested at the end of treatment. DAPI (blue) was used to indicate the stained nucleus, and tdTomato (red) was used to indicate positive light‐dependent recombinase activity. Scale bar, 500 µm. e) Genetic labeling during embryonic development. Pregnant female mice were illuminated with blue light (w/ light) (40 mW cm^−2^, 1 min pulse every 5 min) for 36 h or without blue light (w/o light) stimulation. E18.5 embryos were harvested 48 h after light stimulation. f) Genetic labeling in neonatal mice. Mice at postnatal day 0.5 (P0.5) were illuminated with blue light (w/ light) (40 mW cm^−2^, 1 min pulse every 5 min) for 36 h or without blue light (w/o light) stimulation. In (e) and (f), bright‐field (left) and fluorescence images (middle) from whole animals were acquired using a stereomicroscope. Scale bars, 2 mm. Representative fluorescence images were acquired through sections (right). tdTomato (red) expression was used to indicate positive recombination. DAPI (blue) was used to indicate the stained nucleus. Scale bar, 1 mm.

To further explore the function of ePA‐Cre mice, Ai14 Cre reporter mice^[^ ^22^ ^]^ were crossed with ePA‐Cre mice (**Figure** **5**a). First, the in vitro recombination effect was tested. Fibroblasts were isolated from the tail tissues of double transgenic progenies and exposed to blue light. The results showed that under various durations and intensities of illumination, Cre recombinase activity was exposure time‐ and illumination intensity‐dependent, and 1 min of blue‐light stimulation (1 mW cm^−2^) was sufficient to drive tdTomato expression in vitro (Figure 5b,c). Next, the in vivo recombination effect in various tissues, including the heart, liver, spleen, etc., was evaluated. To stimulate highly efficient recombination, especially in the internal organs, we employed longer illumination time and higher illumination intensity (60 mW cm^−2^, 1 min pulse every 5 min for 48 h). The hair of 6‐week‐old ePA‐Cre: Ai14 double transgenic mice was removed and the mice were exposed to blue light. Then, the tissues were dissected and imaged using a stereomicroscope. The data showed that red fluorescent signaling was observed in all examined tissues, which meant that recombination occurred successfully, and no background activity was detected in the organs without blue light illumination (Figure S6, Supporting Information). Considering that tdTomato signaling might be mainly observed at the surface of tissue through stereomicroscopy and caused by insufficient illumination due to the limited penetration of blue light in different tissues, histological analyses were performed to demonstrate the spatial‐specificity pattern of tdTomato expression. The results showed that blue light penetrated the heart, liver, and kidney to approximately 750–875 µm (Figure 5d), and as expected, the recombination efficiency gradually reduced with increasing tissue depth in the tested tissues (Figure S7, Supporting Information). We found that similar to cell culture, 5‐min illumination could induce tdTomato expression in mouse liver (Figure S8, Supporting Information). Notably, although induced with noninvasive blue light, ePA‐Cre was dramatically activated in deep mouse brain regions (Figure S9a, Supporting Information). When the duration of light exposure increased to 48 h, ePA‐Cre was activated in whole brain tissue indicating a sensitive response to blue light in mouse brain (Figure S9b,c, Supporting Information). To further assess the leaky activity of ePA‐Cre in the brain, we measured tdTomato expression in selected brain regions treated with or without blue light. qRT‐PCR analysis confirmed very low leaky activity in the brain tissues of ePA‐Cre mice (Figure S10, Supporting Information). We also investigated ePA‐Cre‐mediated recombination in mouse embryos and neonatal mice. Red fluorescence could be observed macroscopically from the whole bodies of embryos and pups following blue light exposure. In contrast, no tdTomato signals were detected in the embryos and newborn mice without illumination (Figure 5e,f). Of note, we performed pulse illumination in vivo. Therefore, 48 h of pulse illumination (1 min pulse every 5 min) was approximately 8 h of continuous illumination which was shorter than a previous study which used 16 h of continuous illumination.^[^ ^12^ ^]^ In addition, users can shorten the illumination time via increasing the illumination strength, extending the illumination pulse, or designing new illumination strategies by using an optical fiber^[^ ^15^, ^23^, ^24^ ^]^ or two‐photon.^[^ ^13^ ^]^ In summary, the data above demonstrated that the recombinase activity was activated efficiently and dramatically in various tissues upon blue light induction in ePA‐Cre: Ai14 mice without obvious background activity.

### 2.5 Stringent Light Responsiveness in ePA‐Cre Mice

The results above demonstrated that the recombination activity of ePA‐Cre was stringently activated under blue light induction in embryos and neonatal and adult mice. However, since unexpected dimerization and activity leakage might occur when the intracellular amount of Cre or reporter increases with age,^[^ ^16^ ^]^ we decided to explore whether ePA‐Cre activity was still strictly shut off in transgenic mice under normal housing conditions for long‐term. Representative organs were harvested from 12‐week‐old ePA‐Cre: Ai14 double transgenic mice. No recombination effect was observed in the various tested tissues except the skin, which was easily exposed to normal lighting conditions, confirming the tight control of ePA‐Cre activity in internal organs (Figure S11, Supporting Information). These data suggest that Cre activity was stringently regulated by blue light illumination in ePA‐Cre mice and that long‐term exposure to normal light could gradually activate ePA‐Cre in skin tissue.

### 2.6 Optogenetic Cell Ablation in Mouse Liver

**Figure 6:**
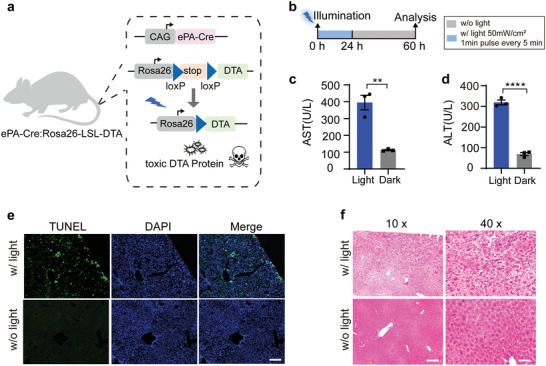
Light‐dependent cell ablation in mouse liver. a) Schematic diagram of light‐dependent activation of DTA expression in ePA‐Cre: Rosa26‐LSL‐DTA mice. DTA became activated while the transcriptional stop cassette flanked by two loxP sites was deleted by ePA‐Cre upon blue‐light illumination. The following experiments were conducted in heterozygous ePA‐Cre: homozygous Rosa26‐LSL‐DTA mice. b) Schematic of the experimental procedure for evaluating cell ablation in vivo. c) Serum AST levels from ePA‐Cre: Rosa26‐LSL‐DTA mice. d) Serum ALT levels from ePA‐Cre: Rosa26‐LSL‐DTA mice. In (c) and (d), the results are shown as the mean ± s.d. Statistical analysis was calculated by Student's *t*‐test; *n* = 3 mice. **P < 0.01, ****P < 0.0001 versus control. e) Representative TUNEL images of liver sections from ePA‐Cre: Rosa26‐LSL‐DTA mice. Apoptotic cells are shown in green. DAPI (blue) indicates the stained nucleus. Scale bar, 200 µm. f) Histological analysis of liver sections with hematoxylin and eosin. Liver sections from illuminated mice presented with severe specific damage to hepatocytes (top). Liver sections from mice without blue light (w/o light) stimulation was normal (bottom). Scale bar, 200 µm for 10× magnification images (left) and 50 µm for 40× magnification images (right).

A major challenge in studying the role of specific cell populations is the lack of versatile cell ablation tools.^[^ ^25^ ^]^ Although chemically inducible Cre strains are widely used to achieve this goal, the temporal and spatial resolution is limited. To demonstrate the ability to perform light‐induced cell ablation, ePA‐Cre mice were crossed with the Rosa26‐LSL‐DTA line,^[^ ^26^ ^]^ which produces the cytotoxic diphtheria toxin fragment A (DTA) gene upon Cre‐induced recombination (**Figure** **6**a). The DTA subunit directly inhibits protein synthesis, leading to cell death via the apoptotic pathway.^[^ ^27^ ^]^ To easily monitor cell damage, liver tissues of 6‐week‐old mice were targeted. The abdomens of double transgenic mice were exposed to blue light or not subjected to light stimulation (Figure 6b). Two days post‐treatment, only mice exposed to blue light exhibited approximately four fold elevation in serum aspartate aminotransferase (AST) and alanine aminotransferase (ALT) levels, two major biomarkers for liver damage, compared with the levels found in control mice (Figure 6c,d). The TUNEL assay revealed that a considerable proportion of hepatocytes underwent apoptosis in light‐treated mice. Moreover, TUNEL‐positive cells were mainly concentrated at the lower edge of the liver, where cells received more photons, indicating that it is a light‐induced phenotype in the mice (Figure 6e). To further confirm the condition of the hepatocytes, liver tissue sections were stained with hematoxylin and eosin (HE). Consistently, we found severe hepatocyte damage in the livers of mice exposed to blue light, whereas liver sections from the control mice displayed a normal phenotype (Figure 6f). In addition to mouse liver, light‐induced cell ablation was also performed in the spleen (Figure S12a, Supporting Information). We also noticed signals of TUNEL‐positive cells in ePA‐Cre mice without illumination, although much less than illuminated mice (Figure S12b, Supporting Information), suggesting the leakage of Cre activity in 12‐week‐old ePA‐Cre mouse skin. It was consistent with the above data showing the Cre leakage in elder mice (Figure S11, Supporting Information). In summary, we concluded that the ePA‐Cre transgenic strain could be used for efficient spatiotemporal cell ablation in multiple tissues and would be a valuable approach for dissecting cell functions.

## 3 Discussion

Spatiotemporal control of in vivo genetic manipulation is critical but challenging for biomedical studies. To date, several elegant photoactivatable recombinases have been developed mainly based on the CRY2‐CIB1^[^ ^10^ ^]^ and Magnets system,^[^ ^28^ ^]^ such as PA‐Cre, PA‐Flp, and PA‐Dre, offering promising approaches for high‐resolution spatiotemporal transgenesis.^[^ ^10^, ^11^, ^12^, ^23^, ^24^ ^]^ In this study, we optimized the PA‐Cre2.0 system leading to ePA‐Cre, which exhibited enhanced efficiency and reduced leakage compared with other tested PA‐Cre systems. A transgenic mouse strain containing the ePA‐Cre system was generated, and the Cre activity was stringently responsive to blue light illumination in multiple mouse tissues. Since ePA‐Cre is driven by a ubiquitous promoter in transgenic mice, genetic recombination could ideally be induced in all cell types in response to light induction.

Stringency is critical for optogenetic tools, especially for stable transgenic animal strains, since stable transgenesis has difficulty titring the number of constructs delivered. A recently generated Magnets‐PA‐Cre KI mouse model exhibited visible background activity in multiple tissues. Although no leakage was observed in newborns, 5–7% spontaneous recombination was observed in the heart, liver, and thymus at 8 weeks of age,^[^ ^16^ ^]^ suggesting that it is a progressive process that could potentially induce much higher leakage when the mice age. To avoid high background activity in transgenic mice, the transgene construct used is most important. In this study, we leveraged an in vivo strategy to evaluate the plasmids and determined that the CRY2‐CIB1 split Cre system exhibited a lower background than the Magnets‐PA‐Cre system (Figure 1), which is similar to previous studies in cell lines.^[^ ^15^ ^]^ Before generation of the transgenic mice, we first confirmed our ePA‐Cre system in Ai14 transgenic reporter mice, as evaluation of endogenous gene recombination is more reliable than transient transgenes. Moreover, we performed functional screening of ePA‐Cre mice via tail vein injection of AAV vectors containing firefly luciferase reporter. These multistep quality controls identified that the ePA‐Cre mouse had the best performance, especially for the great reduction in leakage.

We carefully characterized the light‐dependent recombination activity of ePA‐Cre mice at different developmental stages as well as in various tissues. To our knowledge, this is the first study to show a light‐inducible Cre system for embryonic and postnatal genetic engineering. To our surprise, ePA‐Cre functioned in both entire embryos and neonatal mice (Figure 5e,f). Compared with the most widely used tamoxifen‐inducible Cre‐ER mouse model, the ePA‐Cre mouse has the advantage of genetic modulation in embryos and neonates, since systemic administration of chemical inducers has the potential risk of side effects in pregnant mice; for example, a high dose of tamoxifen induces abortion or can affect normal development.^[^ ^29^ ^]^ Immunofluorescence images revealed that ePA‐Cre triggered robust tdTomato expression approximately 750–875 µm from the surface of some organs, such as the heart, liver, and kidney, but surprisingly, ePA‐Cre could be activated in whole brain tissue (Figure S9, Supporting Information). This suggests that blue light can penetrate mouse skull bone and that ePA‐Cre mice would be a valuable tool for noninvasive brain gene manipulation. If using an implanted optical fiber, we believe that the ePA‐Cre could be activated in a specific position of the brain for sparse labeling through fine tuning of the laser energy, as in our previous report.^[^ ^24^ ^]^ As mosaic analysis in mice is a very important technology,^[^ ^30^ ^]^ ePA‐Cre, which could be activated only in partial cells, could be a valuable tool for positional mosaic analysis, especially for tracing long‐distance cell migration.

The ePA‐Cre mouse also has some limitations. Although no obvious leakage was observed in any tested tissues in young ePA‐Cre mice, significant recombination was detected in the skin at 12 weeks of age due to the long‐term exposure to environmental lights. Inspired by Li‐rtTA knock‐in mice, tight regulation can be achieved in mouse skin under dual control of blue light and doxycycline.^[^ ^31^ ^]^ As ePA‐Cre is driven by a ubiquitous promoter, it could not be used to perform cell type‐specific gene recombination. A dual recombinase strategy with a tissue‐specific promoter could address this limitation, as in our previously developed CALID system, which is a tissue‐specific Cre‐activated light‐inducible Dre recombinase. We also noticed that ePA‐Cre triggered robust tdTomato expression, but the penetration of blue light was limited to reach deep into solid tissues. This problem can be solved by using implantable optical fibers,^[^ ^23^ ^]^ lanthanide‐doped upconversion nanoparticles (UCNPs),^[^ ^32^, ^33^ ^]^ ultra‐photosensitive CRY2 variant, named CRY2^E281A^‐A9,^[^ ^34^ ^]^ or the near‐infrared light system (Bphp1‐Q‐PAS1).^[^ ^35^ ^]^

As a proof‐of‐concept experiment, we used the ePA‐Cre: Rosa26‐LSL‐DTA mouse to achieve light‐induced spatiotemporal cell ablation in the liver. To our knowledge, this is the first study to use light to ablate cells in higher animals. Due to its noninvasive and spatiotemporal specific features, this application would benefit profound developmental, physiological, pathological, and neurological studies. In conclusion, the superior stringency of the ePA‐Cre mouse makes the model a powerful tool for efficient, spatiotemporal, highly tunable, and noninvasive genome engineering for further biological applications.

## 4 Experimental Section

### Plasmid Construction

Codon‐optimized Cre, CIB1, CRY2 (L348F), nMag, and pMag cDNA were synthesized by Genewiz. DNA fragment encoding WPRE was amplified from lentiCRISPR version 2 (Addgene 52961). The DNA cassettes of CRY2‐CIB1‐PA‐Cre and Magnets‐PA‐Cre were constructed in the transposon‐bearing plasmid pT2/BH (Addgene #26557). The reporter plasmids, loxP‐stop‐loxP‐Gluc, and loxP‐stop‐loxP‐Fluc were inserted into the AAV backbone construct under the control of a CMV promoter. All plasmids generated in this study were constructed using ClonExpress MultiS One Step Cloning Kit (Vazyme Biotech co.,ltd. catalog no. C113) and confirmed by Sanger sequencing (Tsingke Biotechnology Co., Ltd).

### Cell Culture

Human embryonic kidney cells (HEK‐293T, ATCC CRL‐3216) were cultured in Dulbecco's modified Eagle's medium (DMEM) supplemented with 10% (v/v) fetal bovine serum (Gibco), 1% (v/v) penicillin and streptomycin (Basalmedia). Fibroblast cells were derived directly from mouse tail tissues as described in the previous study^[^ ^36^ ^]^ and cultured in DMEM supplemented with 0.2% (v/v) primocin (Invitrogen), 20% (v/v) fetal bovine serum (Gibco), and 1% (v/v) penicillin and streptomycin (Basalmedia). All cell lines were cultured at 5% CO_2_ and 37 °C incubation and regularly tested for mycoplasma contamination.

### Luciferase Reporter Assays In Vitro

HEK‐293T cells were seeded into 24‐well plates (corning) at a density of 1.5 × 10^5^ cells per well. The next day, cells were transfected with corresponding plasmid mixtures using polyethylenimine (PEI, Polysciences). CRY2‐CIB1‐PA‐Cre and loxP‐stop‐loxP‐Gluc reporter were both 100 ng per well. 8 h after transfection, the medium was changed. For dark group, the 24‐well plates were wrapped in tinfoil avoiding interference from natural light. For illumination group, cells were placed under the timer‐controlled LED arrays (Figure S13a, Supporting Information) and illuminated with blue light (5 mW cm^−2^, 1 min pulse every 5 min) for 24 h. The light intensity was adjusted by DC Power Supply (HYELEC, HY3005B). Then, the cell culture medium was collected and luciferase activities were measured with the Stop & Glo Reagent (Promega) using a LumiSTAR Omega (BMG LabTech) counter following the manufacturer's instructions.

### Hydrodynamic Liver Injection and Reporter Assays

The abdominal fur of five‐week‐old C57BL/6 mice was removed. These mice were then subjected to intravenous injection of plasmids in proper volume (0.1 mL per mouse weight (g)) of Ringer's solution (147 mm NaCl, 1.13 mm CaCl_2_, 4 mm KCl) within 57 s. For the comparison between CRY2‐CIB1‐PA‐Cre and Magnets‐PA‐Cre or between ePA‐Cre and PA‐Cre2.0, we injected corresponding photoactivatable cre (100ug) and loxP‐stop‐loxP‐Fluc reporter (100ug) plasmids into wild‐type mice. 8 h post injection, the mice were randomly divided into two groups. One group of mice was raised normally without light stimulation as controls. The other group of mice was exposed to pulse illumination (1 min pulse every 5 min) for 16 h which was able to reduce the temperature generated by light source considering the ethics and welfare of laboratory animals. For deeper tissue penetration, the hair around the targeted regions was removed. The customized light source (Guangxi Sanyi Lighting Electronics CO., Ltd, LX‐ZWD60‐3) with 0.5 cm average distance between LEDs, was under the white transparent cage in which mice were kept freely access to food and water (Figure S13b, Supporting Information). Unless otherwise mentioned, this procedure was employed for noninvasive light stimulation in most of in vivo experiments. The light intensity per unit area (mW cm^−2^) at the surface of cage was measured using the power meter (NEWDOON, Aurora‐N121C). Bioluminescence imaging was performed 24 h after hydrodynamic transfection using an IVIS Spectrum instrument (Caliper Life sciences). Five minutes after intravenous injection with 100 µL D‐Luciferin, the mice were anesthetized with isoflurane, and images were captured within 10 min. Ai14 mice (Gt (ROSA)26Sor^tm14(CAG‐tdTomato)Hze^) were used for the endogenous reporter activation experiment. 8 h after hydrodynamic transfection with ePA‐Cre plasmids (100 µg), the mice were randomly divided into two groups, and illuminated from the bottom with blue LED (20 mW cm^−2^, 1 min pulse every 5 min) for 16 h or without blue light stimulation. 72 h after the hydrodynamic injection, the mice were sacrificed by carbon dioxide asphyxiation, and the livers were dissected for imaging using fluorescent stereoscope (Leica) or histological analysis.

### AAV‐Mediated Recombination in Mice

For AAVs production, HEK‐293T cells were seeded in a 10‐cm dish and transfected with three‐plasmid system (AAV‐loxP‐stop‐loxP‐Fluc, 7.5 µg; helper plasmid, 10 µg; AAV8 packaging plasmid, 7.5 µg) for each dish using PEI (90 µL, 1 µg µL^−1^). At 72 h post‐transfection, cells were collected and purified using the iodixanol purification method. The viruses were titrated by qPCR and stored at −80 °C until use. Viral titers were 5 × 10^12^ vg mL^−1^ for AAV‐loxP‐stop‐loxP‐Fluc. For screening founder mice, AAV8 viruses encoding loxP‐stop‐loxP‐Fluc (5 × 10^11^ vg for each mouse) in a total volume of 1 mL PBS were delivered into ePA‐Cre mice through tail vein injection. Before bioluminescence images, the abdominal fur of the mice was removed, and the luciferase activity was first determined 2 weeks after injection. The mice were then exposed to blue LED illumination (20 mW cm^−2^, 1 min pulse every 5 min for 16 h or 30 min). The second luciferase assay was determined 2 days after illumination. The bioluminescence imaging was performed as the above luciferase activity assays in vivo.

### Insertion Sites Identification with NGS Data

The clean DNA‐seq reads were mapped against the library of insertion sequence and the mus musculus reference genome mm10 using bwa v0.7.17‐r1188 (http://bio‐bwa.sourceforge.net), given its high accuracy in short‐read alignment. Split read, of which one end was mapped to the insertion sequence and the other end was mapped to the reference genome was parsed out for further analysis. The insertion site at nucleotide resolution in the genome was investigated on the basis of the breakpoint of these split reads which was measured by BLAT.^[^ ^37^ ^]^ The alignment showed that two reads supported the junction between the left side of the insertion and the genome, and another two reads supported the junction between the right side and the genome. Moreover, the insertion fragment has only one insertion position in the genome, that is, chr3L: 98016848. The WGS data were submitted to the NCBI SRA database under accession number SRR17701607.

### Histology

For paraffin histology, tissues were collected and fixed with 4% PFA overnight. The following day, all fixed tissues were embedded in paraffin, sectioned at 5 µm, and stained with hematoxylin and eosin for pathology. For tdTomato reporter assays, various tissues were fixed, de‐waxed, and stained with DAPI as previously described.^[^ ^24^ ^]^ For embryos and newborn mice, the time of fixation and dehydration was about 36 h. Then the serial sections were cut in 15 µm‐thickness. For TUNEL assays, the liver sections were stained with the DeadEnd Fluorometric TUNEL System (Promega) following the manufacturer's guidelines.

### Western Blot

For immunoblotting, cells were harvested and lysed in Biosharp BL504A RIPA buffer (catalog no. BL504A Tsingke Biotechnology Co., Ltd). Proteins were quantified using Solarbio PC0020 BCA assay kit (catalog no. PC0020‐50T Tsingke Biotechnology Co., Ltd), and then the same amount of total cellular protein (30 µg per well) was separated by 10% SDS‐PAGE. After transmembrane and blocking, the target proteins were detected by primary (anti‐flag, Sigma; anti‐GAPDH, Sigma) and secondary (antimouse IR‐Dye 800CW, Sigma; antirabbit IR‐Dye 680RD, Sigma) antibodies. An Odyssey imager (Li‐COR) was used to visualize labeled immunoblots.

### Serum ALT and AST Assays

Mouse blood was collected using retro‐orbital puncture and incubated statically for 10 min on ice. Then the serum was separated from the blood by centrifugation at 8,000 rpm for 15 min at 4 °C and stored at −80 °C until testing. Both AST and ALT activities were measured by Adicon Clinical.

### Mice

Ai14 mice and Rosa26‐LSL‐DTA mice have been described previously.^[^ ^22^, ^26^ ^]^ The ePA‐Cre transgenic mice were generated by SB transposon‐mediated germline transgenesis as previously described.^[^ ^21^ ^]^ Briefly, the microinjection mixture containing 50 ng µL^−1^ SB100X mRNA and 100 ng µL^−1^ transposon donor plasmid at 1:1 ratio was microinjected into fertilized eggs derived from C57BL/6 mice. All mice used in this study were C57/BL6 strain and kept in a specific pathogen‐free facility. The animal experiments were carried out in strict accordance with the guidelines drafted by the Association for Assessment and Accreditation of Laboratory Animal Care in Shanghai, with protocols approved by the East China Normal University Center for Animal Research (protocol ID: m20200105).

### Statistical Analysis

All data were shown as mean ± s.d and statistical significance was determined with two‐tailed paired Student's *t*‐test. ns, not significant; ∗*P* < 0.05; ∗∗*P* < 0.01; ∗∗∗*P* < 0.001; ∗∗∗∗*P* < 0.0001, when compared with control. Unless otherwise mentioned, *n* = 3 independent experiments in vitro experiments. For the animal experiments, each treatment group consisted of randomly selected mice (*n* = 3). Bioluminescence signal of livers was measured using Living Image 4.4 software (PerkinElmer/Caliper Life Sciences). Fluorescence images derived from a confocal microscope were analyzed using LASX software. Prism 8.0 software (GraphPad) was used for statistical analysis.

## Conflict of Interest

The authors have submitted a patent application (Application Number: 201910746260.6) based on the results reported in this study.

## Author Contributions

H.L. and Y.W. contributed equally to this work. H.L., L.W., and D.L. designed the experiments. H.L., Y.W., Y.Q., X.L., Y.G., Me.L., and S.H. performed the experiments. D.Z., L.L., L.H., X.M., Mi.L., and X.Z. analyzed the data. H.L. and D.L. wrote the manuscript with input from all the authors. D.L. supervised the research.

## Supporting information

Supporting Information

## Data Availability

The data that support the findings of this study are available in the supplementary material of this article.

## References

[1] G. Meinke , A. Bohm , J. Hauber , M. T. Pisabarro , F. Buchholz , Chem. Rev. 2016, 116, 12785. 27163859 10.1021/acs.chemrev.6b00077

[2] B. H. Weinberg , J. H. Cho , Y. Agarwal , N. T. H. Pham , L. D. Caraballo , M. Walkosz , C. Ortega , M. Trexler , N. Tague , B. Law , W. K. J. Benman , J. Letendre , J. Beal , W. W. Wong , Nat. Commun. 2019, 10, 4845. 31649244 10.1038/s41467-019-12800-7 PMC6813296

[3] E. Casanova , S. Fehsenfeld , T. Lemberger , D. R. Shimshek , R. Sprengel , T. Mantamadiotis , Genesis 2002, 34, 267. 10.1002/gene.10153 12395386

[4] K. Schönig , F. Schwenk , K. Rajewsky , H. Bujard , Nucleic Acids Res. 2002, 30, 134e. 10.1093/nar/gnf134 PMC137989 12466566

[5] N. Jullien , I. Goddard , S. Selmi‐Ruby , J.‐L. Fina , H. Cremer , J.‐P. Herman , PLoS One 2007, 2, e1355. 18159238 10.1371/journal.pone.0001355 PMC2131782

[6] R. Sando , K. Baumgaertel , S. Pieraut , N. Torabi‐Rander , T. J. Wandless , M. Mayford , A. Maximov , Nat. Methods 2013, 10, 1085. 24056874 10.1038/nmeth.2640 PMC3947879

[7] X. Tian , B. Zhou , J. Biol. Chem. 2021, 296, 100509. 33676891 10.1016/j.jbc.2021.100509 PMC8050033

[8] J. Wu , M. Wang , X. Yang , C. Yi , J. Jiang , Y. Yu , H. Ye , Nat. Commun. 2020, 11, 3708. 32709899 10.1038/s41467-020-17530-9 PMC7381682

[9] S. T. Yen , K. A. Trimmer , N. Aboul‐Fettouh , R. D. Mullen , J. C. Culver , M. E. Dickinson , R. R. Behringer , G. T. Eisenhoffer , Dev. Dyn. 2020, 249, 1394. 32745301 10.1002/dvdy.232 PMC7931845

[10] M. J. Kennedy , R. M. Hughes , L. A. Peteya , J. W. Schwartz , M. D. Ehlers , C. L. Tucker , Nat. Methods 2010, 7, 973. 21037589 10.1038/nmeth.1524 PMC3059133

[11] A. Taslimi , B. Zoltowski , J. G. Miranda , G. P. Pathak , R. M. Hughes , C. L. Tucker , Nat. Chem. Biol. 2016, 12, 425. 27065233 10.1038/nchembio.2063 PMC4871718

[12] F. Kawano , R. Okazaki , M. Yazawa , M. Sato , Nat. Chem. Biol. 2016, 12, 1059. 27723747 10.1038/nchembio.2205

[13] S. Yao , P. Yuan , B. Ouellette , T. Zhou , M. Mortrud , P. Balaram , S. Chatterjee , Y. Wang , T. L. Daigle , B. Tasic , X. Kuang , H. Gong , Q. Luo , S. Zeng , A. Curtright , A. Dhaka , A. Kahan , V. Gradinaru , R. Chrapkiewicz , M. Schnitzer , H. Zeng , A. Cetin , Nat. Methods 2020, 17, 422. 32203389 10.1038/s41592-020-0774-3 PMC7135964

[14] H. Duplus‐Bottin , M. Spichty , G. Triqueneaux , C. Place , P. E. Mangeot , T. Ohlmann , F. Vittoz , G. Yvert , Elife 2021, 10, e61268. 33620312 10.7554/eLife.61268 PMC7997657

[15] K. Meador , C. L. Wysoczynski , A. J. Norris , J. Aoto , M. R. Bruchas , C. L. Tucker , Nucleic Acids Res. 2019, 47, e97. 31287871 10.1093/nar/gkz585 PMC6753482

[16] K. Yoshimi , Y. Yamauchi , T. Tanaka , T. Shimada , M. Sato , T. Mashimo , Lab. Invest. 2021, 101, 125. 32892213 10.1038/s41374-020-00482-5

[17] K. Morikawa , K. Furuhashi , C. de Sena‐Tomas , A. L. Garcia‐Garcia , R. Bekdash , A. D. Klein , N. Gallerani , H. E. Yamamoto , S.‐H. E. Park , G. S. Collins , F. Kawano , M. Sato , C.‐S. Lin , K. L. Targoff , E. Au , M. C. Salling , M. Yazawa , Nat. Commun. 2020, 11, 2141. 32358538 10.1038/s41467-020-16030-0 PMC7195411

[18] J. L. Jankowsky , H. H. Slunt , T. Ratovitski , N. A. Jenkins , N. G. Copeland , D. R. Borchelt , Biomol. Eng. 2001, 17, 157. 11337275 10.1016/s1389-0344(01)00067-3

[19] F. Liu , Y. K. Song , D. Liu , Gene Ther. 1999, 6, 1258. 10455434 10.1038/sj.gt.3300947

[20] R. Zufferey , J. E. Donello , D. Trono , T. J. Hope , J. Virol. 1999, 73, 2886. 10074136 10.1128/jvi.73.4.2886-2892.1999 PMC104046

[21] Z. Ivics , L. Mátés , T. Y. Yau , V. Landa , V. Zidek , S. Bashir , O. I. Hoffmann , L. Hiripi , W. Garrels , W. A. Kues , Z. Bösze , A. Geurts , M. Pravenec , T. Rülicke , Z. Izsvák , Nat. Protoc. 2014, 9, 773. 24625778 10.1038/nprot.2014.008

[22] L. Madisen , T. A. Zwingman , S. M. Sunkin , S. W. Oh , H. A. Zariwala , H. Gu , L. L. Ng , R. D. Palmiter , M. J. Hawrylycz , A. R. Jones , E. S. Lein , H. Zeng , Nat. Neurosci. 2010, 13, 133. 20023653 10.1038/nn.2467 PMC2840225

[23] H. Jung , S.‐W. Kim , M. Kim , J. Hong , D. Yu , J. H. Kim , Y. Lee , S. Kim , D. Woo , H.‐S. Shin , B. O. Park , W. Do Heo , Nat. Commun. 2019, 10, 314. 30659191 10.1038/s41467-018-08282-8 PMC6338782

[24] H. Li , Q. Zhang , Y. Gu , Y. Wu , Y. Wang , L. Wang , S. Feng , Y. Hu , Y. Zheng , Y. Li , H. Ye , B. Zhou , L. Lin , M. Liu , H. Yang , D. Li , Proc. Natl. Acad. Sci. U. S. A. 2020, 117, 33426. 33318209 10.1073/pnas.2003991117 PMC7777003

[25] F. Liu , S. Dai , D. Feng , X. Peng , Z. Qin , A. C. Kearns , W. Huang , Y. Chen , S. Ergün , H. Wang , J. Rappaport , E. C. Bryda , A. Chandrasekhar , B. Aktas , H. Hu , S. L. Chang , B. Gao , X. Qin , Cell. Mol. Life Sci. 2019, 76, 4725. 31359086 10.1007/s00018-019-03243-w PMC6858955

[26] D. Wang , C. Cai , X. Dong , Q. C. Yu , X.‐O. Zhang , L. Yang , Y. A. Zeng , Nature 2015, 517, 81. 25327250 10.1038/nature13851

[27] R. D. Palmiter , R. R. Behringer , C. J. Quaife , F. Maxwell , I. H. Maxwell , R. L. Brinster , Cell 1987, 50, 435. 3649277 10.1016/0092-8674(87)90497-1

[28] F. Kawano , H. Suzuki , A. Furuya , M. Sato , Nat. Commun. 2015, 6, 6256. 25708714 10.1038/ncomms7256

[29] N. Ved , A. Curran , F. M. Ashcroft , D. B. Sparrow , Lab. Anim. 2019, 53, 630. 31248325 10.1177/0023677219856918 PMC6900213

[30] H. Zong , J. S. Espinosa , H. H. Su , M. D. Muzumdar , L. Luo , Cell 2005, 121, 479. 15882628 10.1016/j.cell.2005.02.012

[31] F. Li , Z. Lu , W. Wu , N. Qian , F. Wang , T. Chen , Cell Res. 2019, 29, 862. 31366989 10.1038/s41422-019-0209-9 PMC6796910

[32] S. Chen , A. Z. Weitemier , X. Zeng , L. He , X. Wang , Y. Tao , A. J. Y. Huang , Y. Hashimotodani , M. Kano , H. Iwasaki , L. K. Parajuli , S. Okabe , D. B. L. Teh , A. H. All , I. Tsutsui‐Kimura , K. F. Tanaka , X. Liu , T. J. McHugh , Science 2018, 359, 679. 29439241 10.1126/science.aaq1144

[33] N. T. Nguyen , K. Huang , H. Zeng , J. Jing , R. Wang , S. Fang , J. Chen , X. Liu , Z. Huang , M. J. You , A. Rao , Y. Huang , G. Han , Y. Zhou , Nat. Nanotechnol. 2021, 16, 1424. 34697491 10.1038/s41565-021-00982-5 PMC8678207

[34] S. Kim , T. Kyung , J.‐H. Chung , N. Kim , S. Keum , J. Lee , H. Park , H. M. Kim , S. Lee , H.‐S. Shin , W. Do Heo , Nat. Commun. 2020, 11, 210. 31924789 10.1038/s41467-019-14005-4 PMC6954201

[35] T. A. Redchuk , E. S. Omelina , K. G. Chernov , V. V Verkhusha , Nat. Chem. Biol. 2017, 13, 633. 28346403 10.1038/nchembio.2343 PMC6239862

[36] M. Khan , S. Gasser , J. Visualized Exp. 2016, 107, e53565. 10.3791/53565 PMC4781275 26779653

[37] W. J. Kent , Genome Res. 2002, 12, 656. 11932250 10.1101/gr.229202 PMC187518

