# Correction to “Stable Transgenic Mouse Strain with Enhanced Photoactivatable Cre Recombinase for Spatiotemporal Genome Manipulation”

**DOI:** 10.1002/advs.202406227

**Published:** 2024-07-02

Li, H., Wu, Y., Qiu, Y., Li, X., Guan, Y., Cao, X., Liu, M., Zhang, D., Huang, S., Lin, L., et al. (2022). Stable Transgenic Mouse Strain with Enhanced Photoactivatable Cre Recombinase for Spatiotemporal Genome Manipulation. *Adv. Sci. 9*(e): e2201352.

https://doi.org/10.1002/advs.202201352

In the original article, there is a typo of Grant number in the Acknowledgements session. The grant number “21CJ1402200” should be replaced as “21JC1402200”.

The corrected contents are provided as follows: The authors are grateful to Shaochuang Liu (Nanjing University) for his technical assistance with timer‐controlled LED devices and the support of the East China Normal University Public Platform for innovation (011). The authors thank the Materials Characterization Center of East China Normal University for helping with fluorescence imaging. This work was partially supported by grants from the National Key R&D Program of China (2019YFA0802802 and 2019YFA0110802), the National Natural Science Foundation of China (No.32025023, No. 32101194), the China Postdoctoral Science Foundation (BX2021104), and grants from the Shanghai Municipal Commission for Science and Technology (21JC1402200, 20140900200), the Innovation Program of Shanghai Municipal Education Commission (2019‐01‐07‐00‐05‐E00054), and the Fundamental Research Funds for the Central Universities, Project of Shanghai Municipal Science and Technology Commission (20MC1920400).

We apologize for this error.

