# Supplementary material for: Stable Transgenic Mouse Strain with Enhanced Photoactivatable Cre Recombinase for Spatiotemporal Genome Manipulation
Source: Adv Sci (Weinh). 2022 Oct 20;9(34):2201352. doi: 10.1002/advs.202201352 (PMC9731692; doi:10.1002/advs.202201352)
Supplement: Supplementary file 1 — Supporting Information [file ADVS-9-2201352-s001.pdf]

ADVANCED SCIENCE
Open Access

Supporting Information

for *Adv. Sci.*, DOI 10.1002/advs.202201352

Stable Transgenic Mouse Strain with Enhanced Photoactivatable Cre Recombinase for Spatiotemporal Genome Manipulation

Huiying Li, Yingyin Wu, Yuhao Qiu, Xinru Li, Yuting Guan, Xiya Cao, Meizhen Liu, Dan Zhang, Sijie Huang, Longnian Lin, Lijian Hui, Xueyun Ma, Mingyao Liu, Xueli Zhang, Liren Wang* and Dali Li**

Supporting Information

Stable transgenic mouse strain with enhanced photoactivatable Cre recombinase for spatiotemporal genome manipulation

Huiying Li[1,2,†], Yingyin Wu [1,†], Yuhao Qiu[1], Xinru Li[1], Yuting Guan[1], Xiya Cao[1],Meizhen Liu[1], Dan Zhang[1], Sijie Huang[3], Longnian Lin[3], Lijian Hui[4], Xueyun Ma[1], Mingyao Liu[1], Xueli Zhang[2,], Liren Wang[1,]*, Dali Li[1,]**

*Corresponding author. Email: lejing1996@aliyun.com, lrwang@bio.ecnu.edu.cn and dlli@bio.ecnu.edu.cn

This PDF file includes:

Supplementary Figures 1-13

Supplementary Table 1

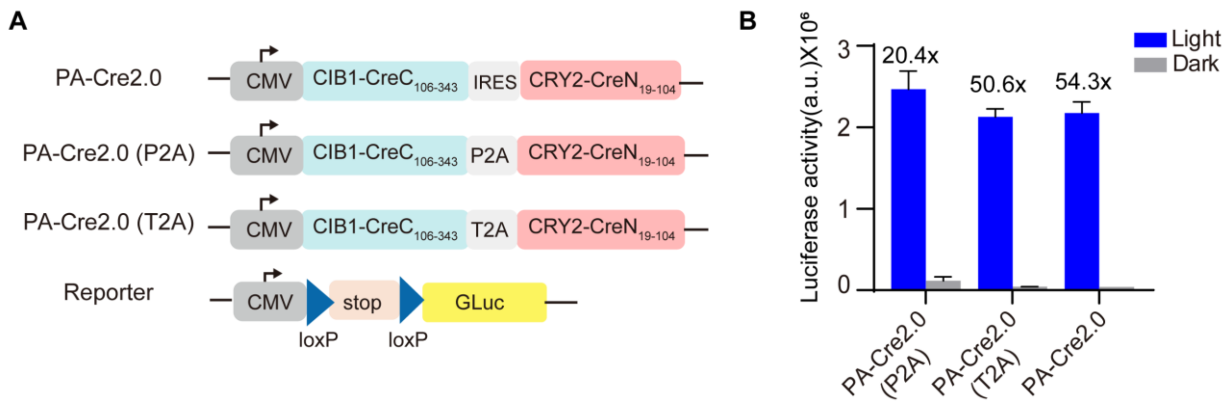

Figure S1 Comparison of the CRY2-CIB1-PA-Cre activity of constructs with variant 2A peptides. (**a**) Schematic of CRY2-CIB1-PA-Cre with two self-cleaving 2A peptides (P2A and T2A) and the Cre-dependent Gaussia luciferase reporter (loxP-stop-loxP-Gluc). (**b**) HEK-293T cells were transfected with the indicated photoactivatable Cre recombinase separately along with a reporter, then exposed to blue light (5 mW/cm^2, 1 min pulse every 5 min) or kept in the dark. Luciferase activity was determined 24 h after the first illumination. The results are shown as the mean ± s.d.; n = 3 independent experiments.

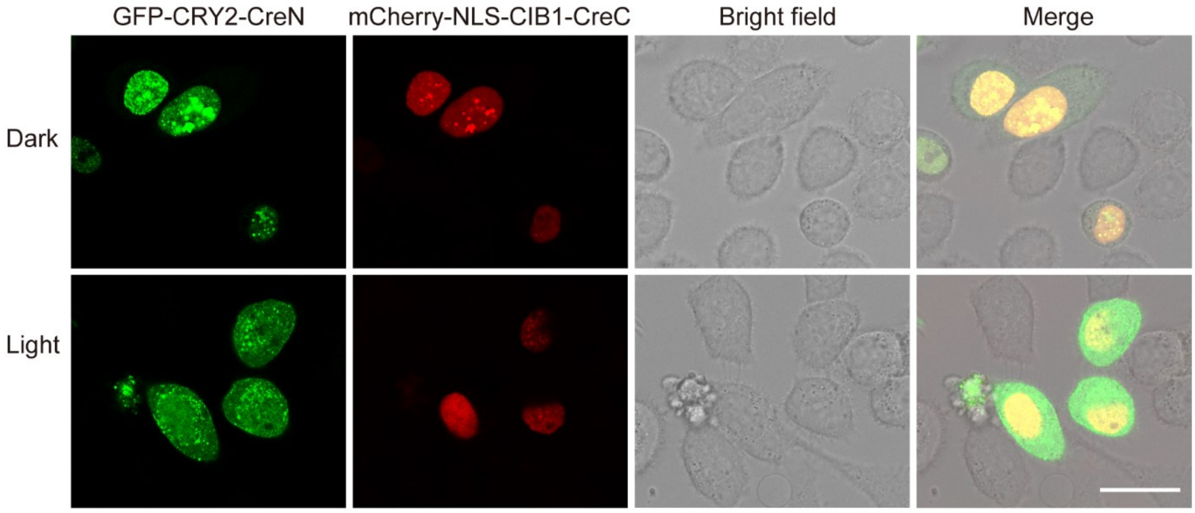

Figure S2 Cellular localization of ePA-Cre compoments. Hela cells were transfected with EGFP-CRY2-CreN (ePA-CreN) and mCherry-NLS-CIB1-CreC (ePA-CreC) constructs. 8 h post-transfection, the cells were exposed to blue light for 24 h or kept in the dark. Flourescent images were obtained through confocal microscopy. EGFP (green) and mCherry (red) signals were used to indicate the distribution of ePA-CreN and ePA-CreC, respectively. Scale bar, 25 μm.

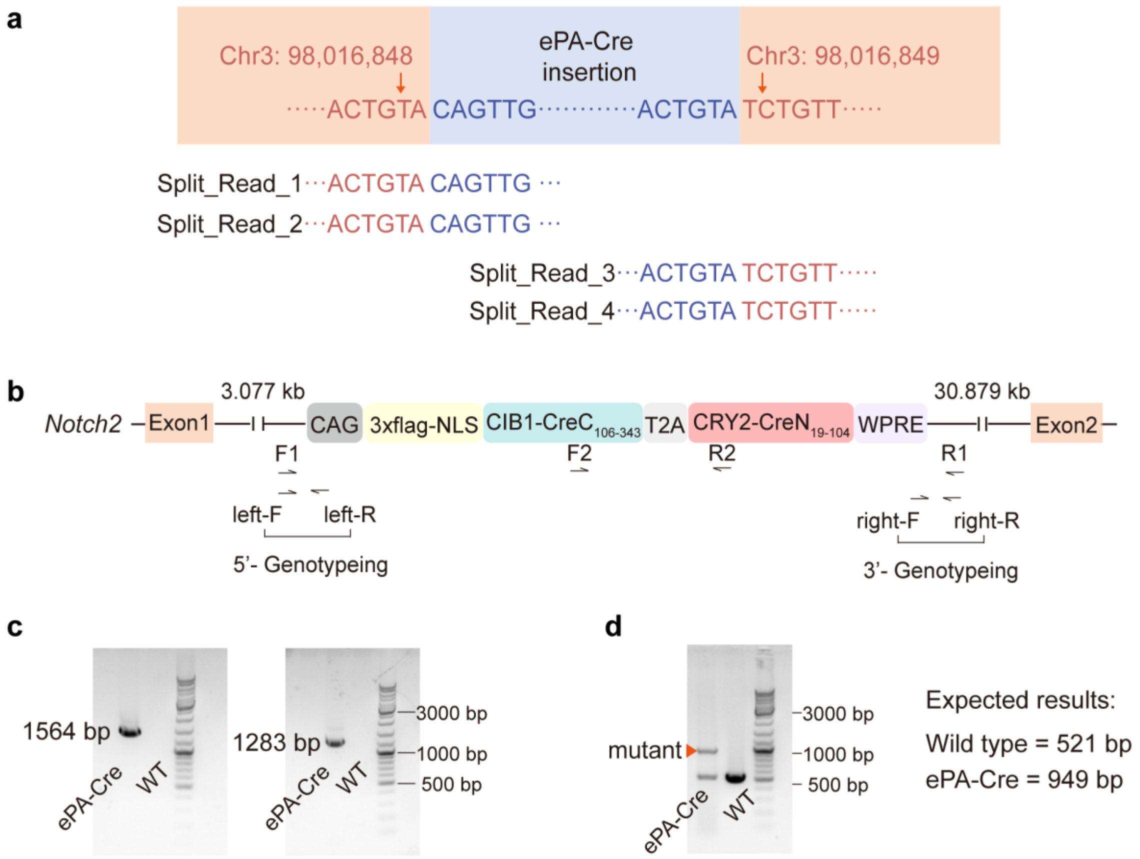

Figure S3 Whole genome sequencing and PCR validation of the ePA-Cre targeting site. (**a**) Four split reads that were used in whole genome sequencing. The sequence mapped to the insertion sequence is highlighted in blue, and the sequence mapped to the reference genome is highlighted in red. (**b**) Primers for PCR amplification of the ePA-Cre targeting site. Primers of left-F/left-R and right-F/right-R spanning the junctions between genomic DNA and the ePA-Cre targeted sequences were used to validate the correct insertion. Primers for F1/R1 and F2/R2 were used for genotyping analysis of ePA-Cre mice. (**c**) Representative electrophoresis images for validation of ePA-Cre insertion site. The insertion site was identified by the 1564 bp (5') and 1283 bp (3') PCR products as marked. (**d**) Representative electrophoresis images of the genotyping results from ePA-Cre offspring. The C57 WT allele was used as a control and is marked by the 521 bp PCR product (F1/R1). The ePA-Cre allele is identified by the 949 bp PCR product (F2/R2).

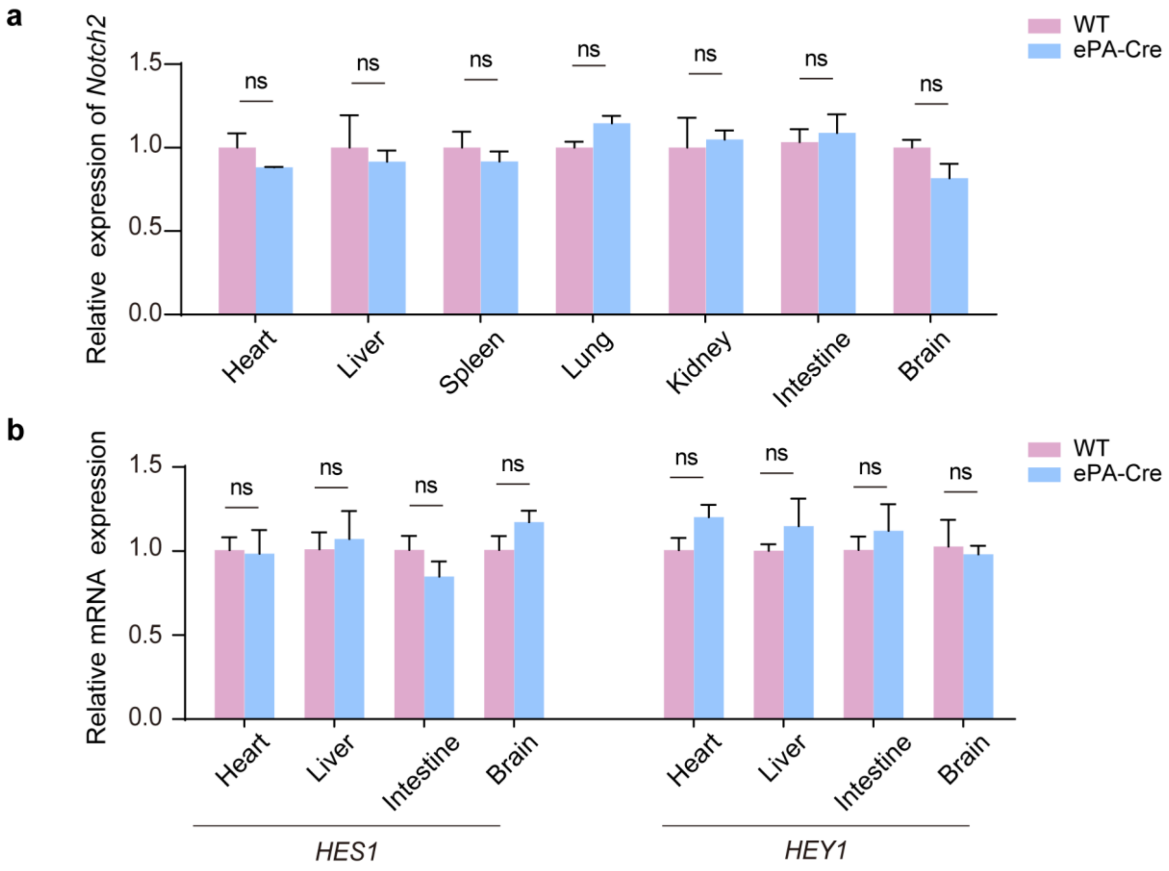

Figure S4 mRNA levels of *Notch2* and its downstream target genes in multiple tissues of ePA-Cre mice. The relative mRNA expression of *Notch2* (**a**) and *Notch2* target genes (*HES1* and *HEY1*) (**b**) in variant tissues of ePA-Cre mice compared with those of WT controls. The values were normalized to GAPDH. The value of WT mice was set to 1. The results are shown as the mean ± s.d. Statistical analysis was performed with Student's *t* test; n = 6 from two mice; n.s., not significant.

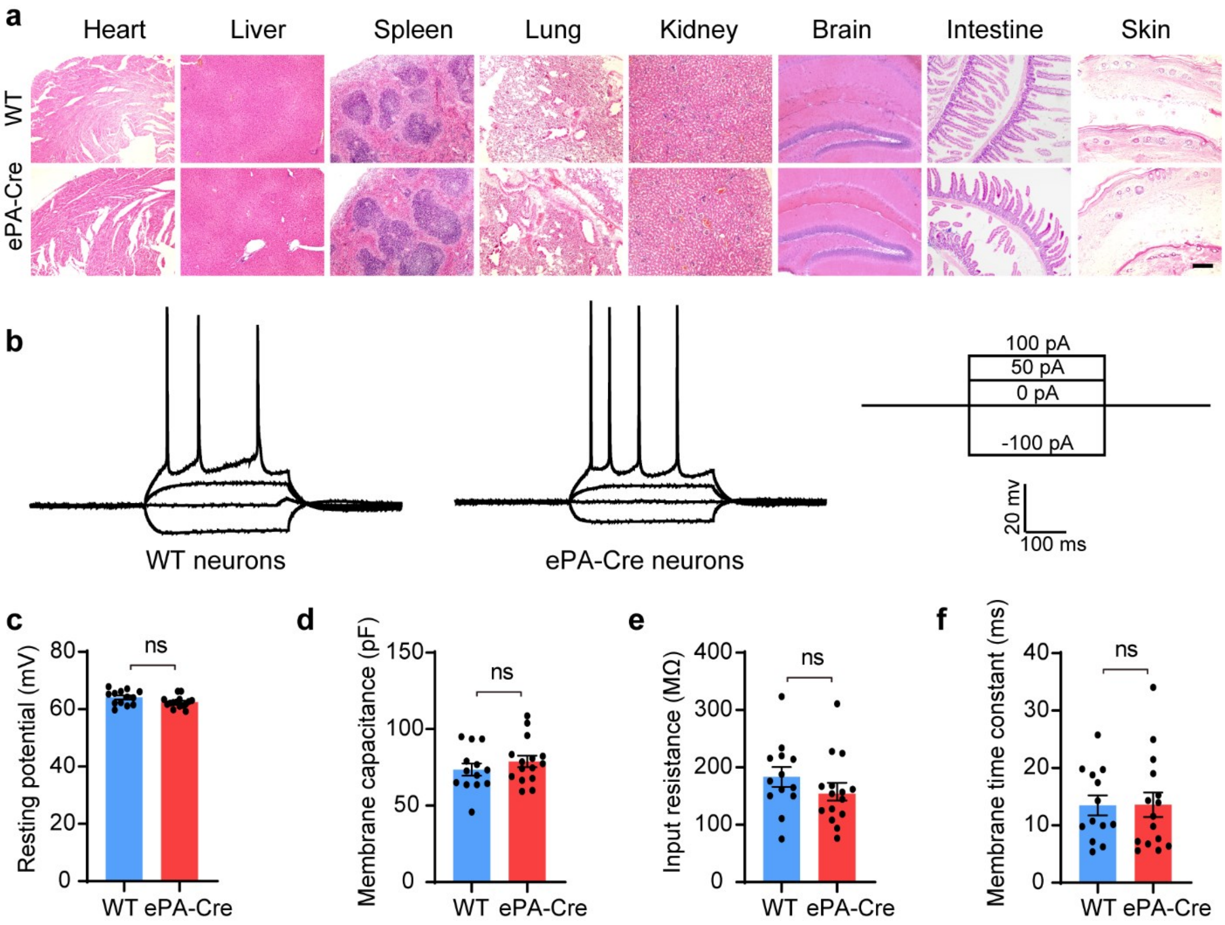

Figure S5 Analysis of cellular morphology and physiology in ePA-Cre mice. (**a**) Sections stained with hematoxylin-eosin from various tissues of wild-type and ePA-Cre mice. Scale bars, 200 μm. (**b**) Representative traces of evoked action potentials from wild-type (left) and ePA-Cre-expressing neurons (right). The injected currents were 100, 50, 0 and −100 pA for 100 ms. (**c**-**f**) Electrophysiological characterization of wild-type and ePA-Cre-expressing hippocampal neurons from acute slices. Resting potential (c), membrane capacitance (d), input resistance (e) and membrane time constant (f). The results are shown as the mean ± s.d. Statistical analysis was performed with Student's *t test*; *n* =12 neurons from three wild-type mice and *n* = 15 neurons from three ePA-Cre mice. n.s., not significant.

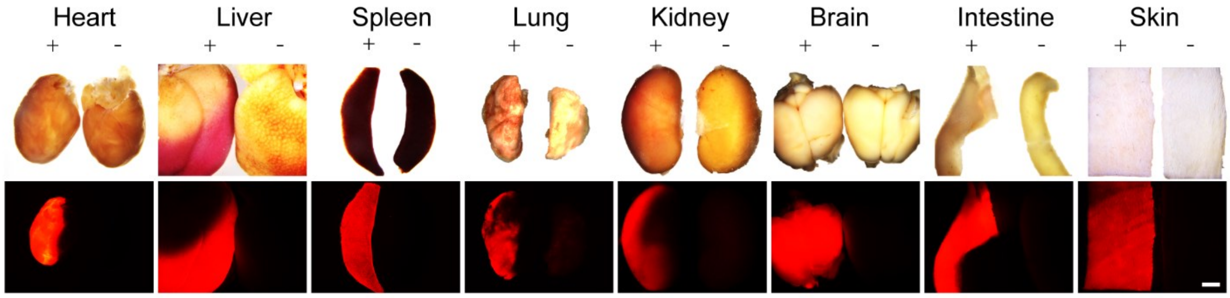

Figure S6 ePA-Cre-induced genetic labelling in various tissues. Representative bright field (top) and fluorescence images (bottom) were acquired using a stereomicroscope. The fluorescence signal of tdTomato is shown in red. Scale bars, 2 mm. +, with blue light; -, without light stimulation.

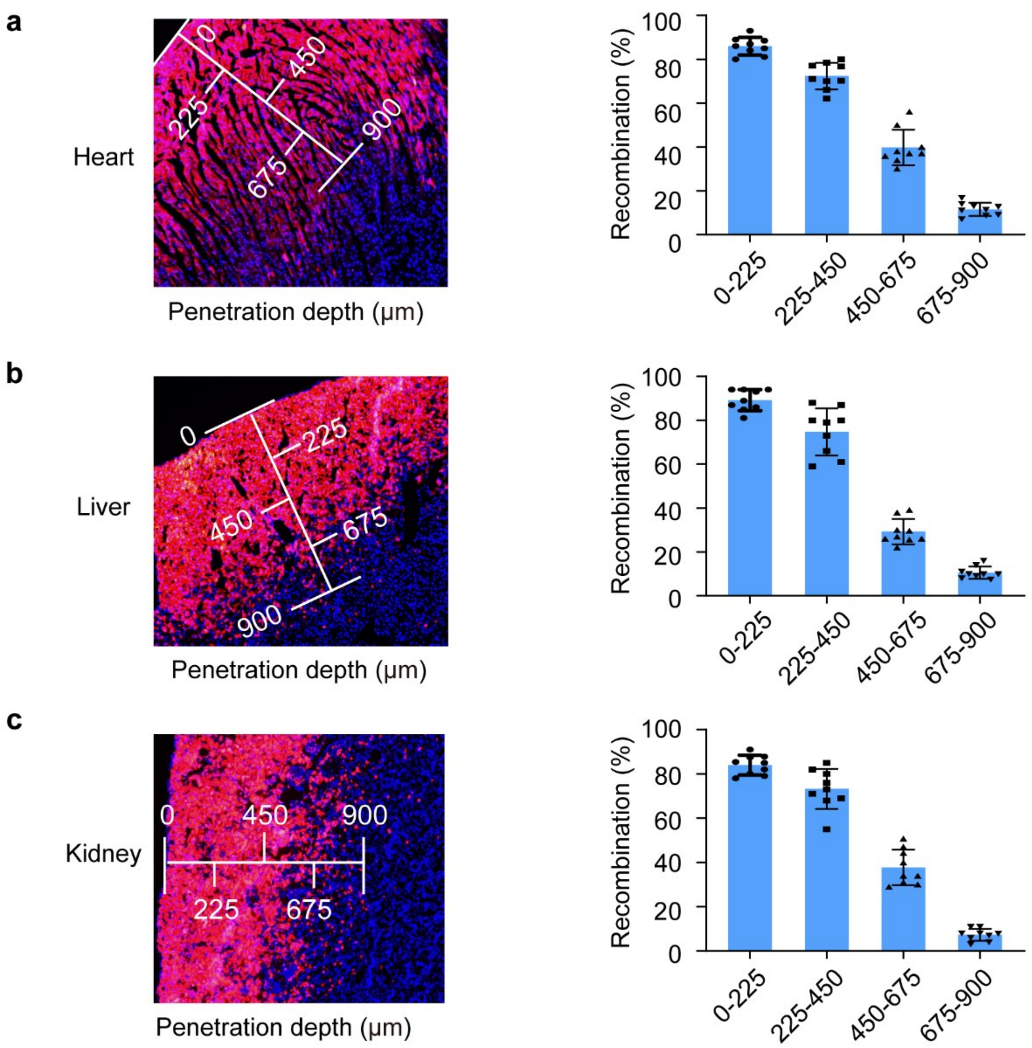

Figure S7 Quantification of ePA-Cre recombination efficiency at different penetration depths in various tissues. The average ratio of tdTomato-expressing areas to total tissue regions of the heart (a), liver (b) and kidney (c) was calculated as recombination efficiency. The results are shown as the mean ± SD; *n* =9 images from three mice.

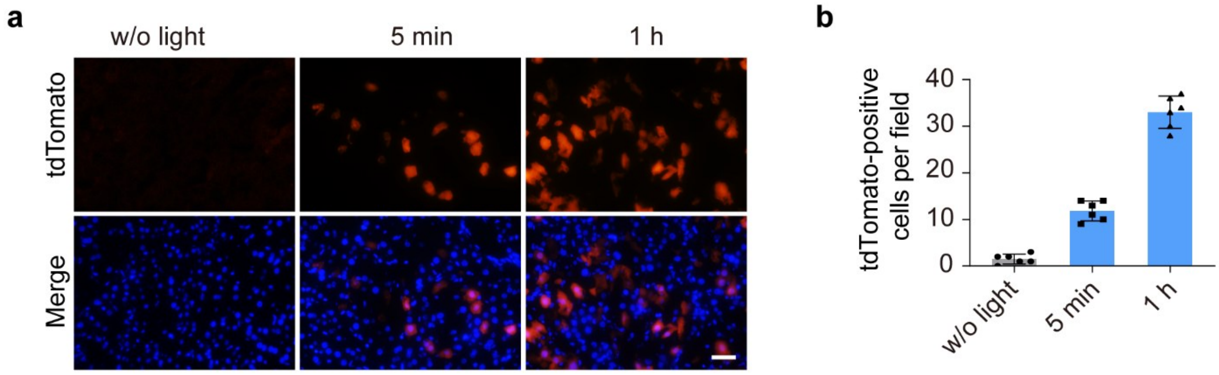

Figure S8 ePA-Cre activation with shorter periods of illumination in mouse liver. (**a**) Representative images of liver tissues from ePA-Cre:Ai14 mice treated without or with continuous illumination (20 mW/cm^2) for 5 min or 1 h. DAPI (blue) indicates the nucleus, tdTomato (red) expression was used to indicate positive recombination. Scale bar, 50 µm. (**b**) Quantification of tdTomato-positive cells per field shown in (a). The results are shown as the mean ± SD; n = 12 images from three mice.

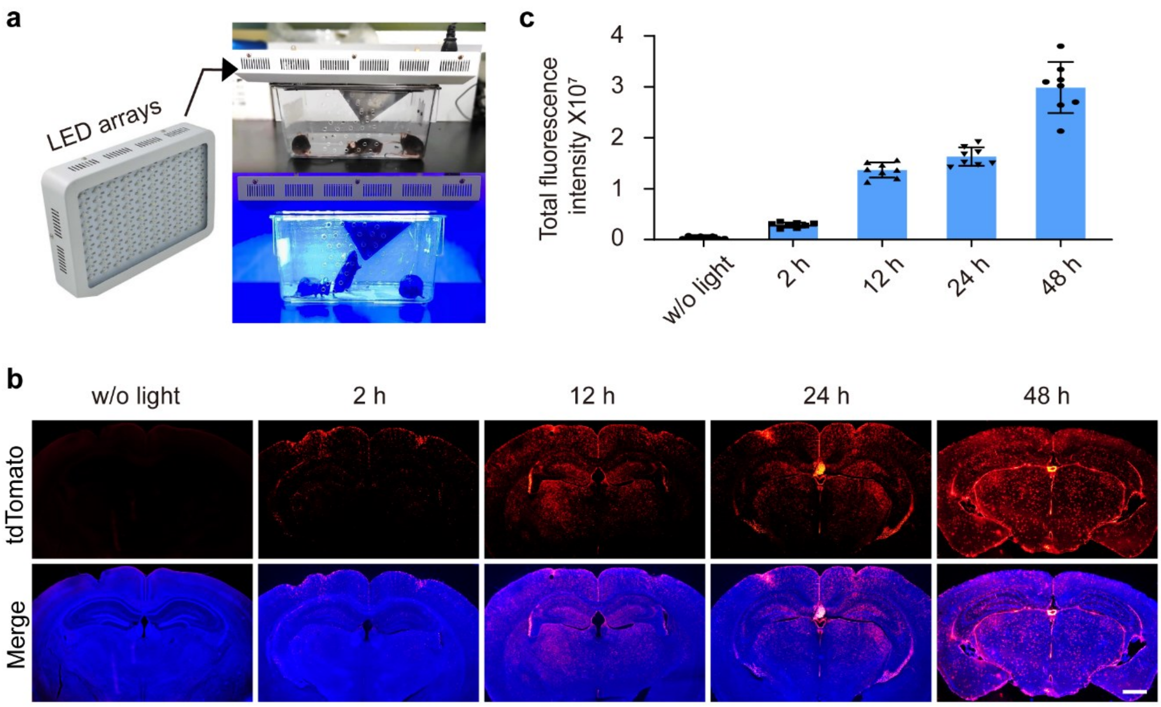

Figure S9 Characterization of ePA-Cre activity in the mouse brain. (**a**) Noninvasive LED illumination activates ePA-Cre in mouse brain without or with illumination (60 mW/cm^2, 1 min pulse every 5 min). (**b**) Representative fluorescence images of mouse brain illuminated for the indicated durations. DAPI (blue) signals indicate the stained nucleus, tdTomato (red) expression was used to indicate positive recombination. Scale bar, 1 mm. (**c**) Quantification of the total red fluorescence intensity of tdTomato expression in the whole brain tissue slides shown in (b). The results are shown as the mean ± SD; n =8 images from two mice.

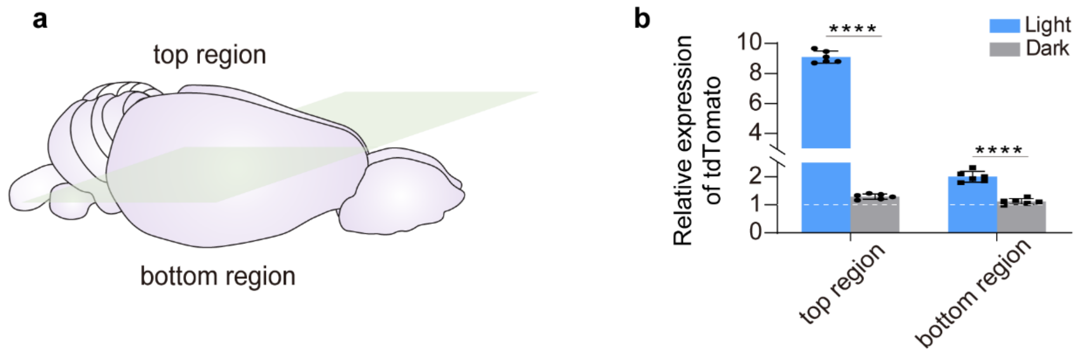

Figure S10 mRNA expression level of tdTomato in ePA-Cre mice at selected brain regions. (**a**) Schematic depicting the cutting of the mouse brain along the indicated plane. (**b**) Relative tdTomato mRNA expression determined by RT-PCR using RNA extracted from the selected brain region shown in (a). ePA-Cre: Ai14 mice were treated with or without noninvasive LED illumination (60 mW/cm^2, 1 min pulse every 5 min for 24 h). The values were normalized to GAPDH. The tdTomato expression value obtained for the brains of ePA-Cre mice was set to 1. The results are shown as the mean ± s.d. Statistical analysis was performed with Student's *t* test; n = 6 from two mice; ****$P < 0.0001$ versus dark control.

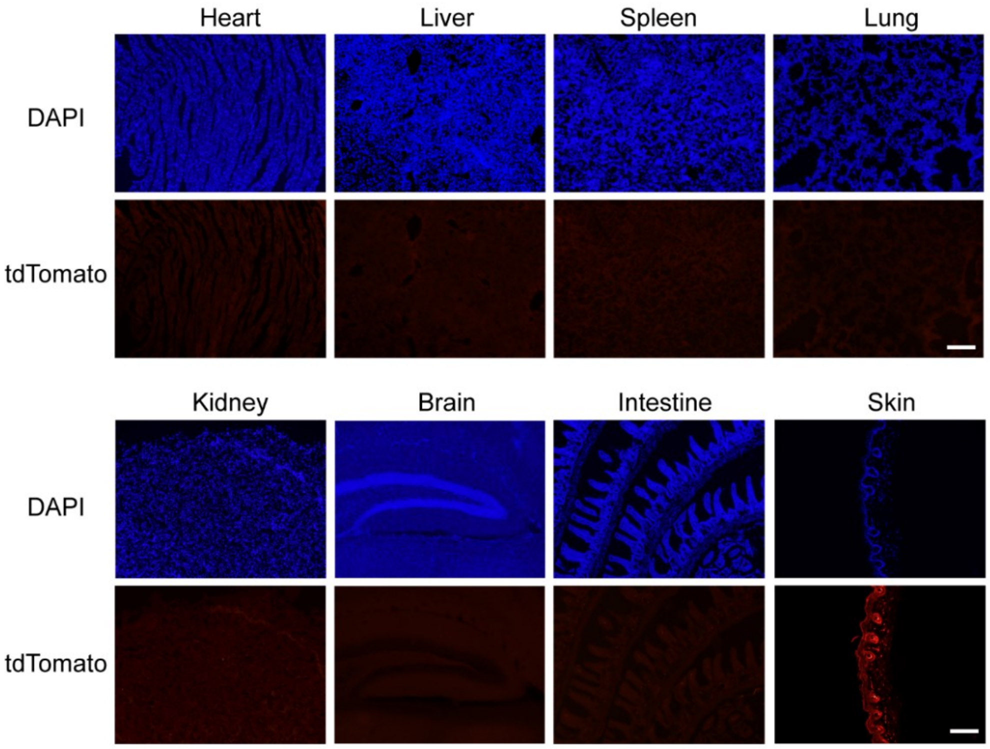

Figure S11 Leak activities in ePA-Cre: Ai14 mice under natural conditions. Representative fluorescence images of different organs from 12-week-old ePA-Cre: Ai14 mice. DAPI (blue) indicates the stained nucleus, tdTomato (red) expression was used to indicate positive recombination. Scale bar, 200 µm.

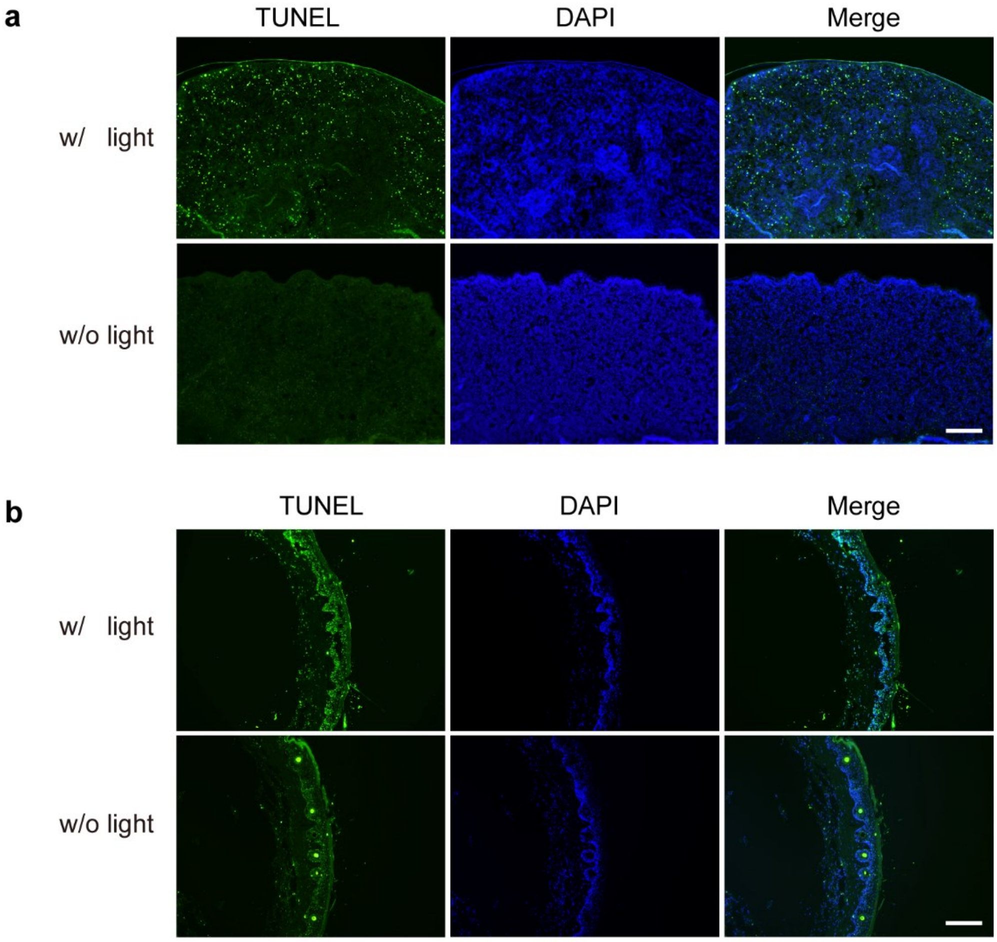

Figure S12 Tissue-specific cell ablation in ePA-Cre: Rosa26-LSL-DTA mice. Representative fluorescence images of spleen (**a**) and skin (**b**) sections of ePA-Cre: Rosa26-LSL-DTA mice treated with blue light (w/ light) or without blue light (w/o light) stimulation. Apoptotic cells were detected by the TUNEL assay (green). DAPI (blue) indicates the stained nucleus. Scale bar, 200 μm.

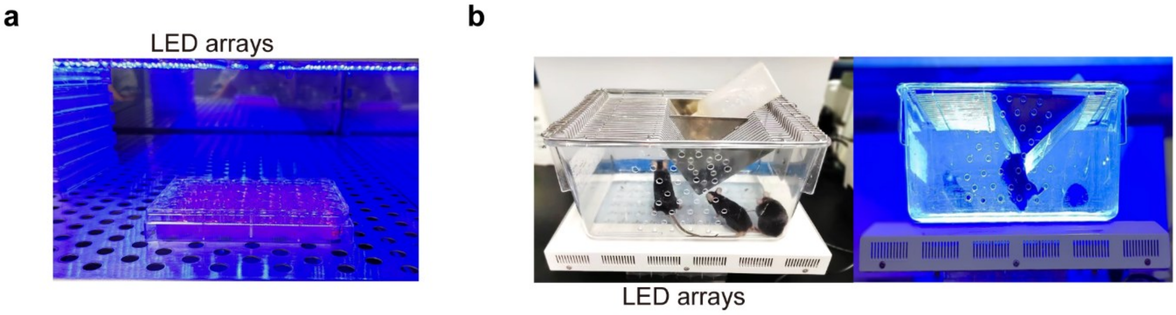

Figure S13 The light sources used for illumination *in vitro* and *in vivo*. (**a**) Timer controlled LED arrays were mounted in the cell incubator for cell illumination. (**b**) Timer controlled LED arrays were used for noninvasive light stimulation in living mice.

Supplementary Table 1. Primers used in this study

Primer name	Primer sequence (5'-3')
left-F	GGAGCTGTCAACCCATCTGT
left-R	CCAAGTGGGCAGTTTACCGT
right-F	GACGTCCTTCTGCTACGTCC
right-R	CACGTCTTCACAGGATGCCA
F1	AACCAGCCATTCCAGCAGTT
R1	TTTTCTGGCTCCCGGCTTAG
F2	ATCCCCTGAGCTGCTTCAAC
R2	TCCCTCCTGAACCAGACGAT
tdTomato-F	GACACCAAGCTGGACATCAC
tdTomato-R	ACCTTGAAGCGCATGAACTC
Notch2-F	CCACCTGCAATGACTTCATCGG
Notch2-R	TCGATGCAGGTGCCTCCATTCT
HES1-F	GGAAATGACTGTGAAGCACCTCC
HES1-R	GAAGCGGGTCACCTCGTTCATG
HEY1-F	CCAACGACATCGTCCCAGGTTT
HEY1-R	CTGCTTCTCAAAGGCACTGGGT
GAPDH-F	GACTTCAACAGCAACTCCCAC
GAPDH-R	TCCACCACCCTGTTGCTGTA
